# Giant Stress-Impedance Effect in CoFeNiMoBSi Alloy in Variation of Applied Magnetic Field

**DOI:** 10.3390/ma14081919

**Published:** 2021-04-12

**Authors:** Piotr Gazda, Michał Nowicki

**Affiliations:** 1 Warsaw University of Technology, Institute of Metrology and Biomedical Engineering, 02-525 Warsaw, Poland; 2 Łukasiewicz Research Network—Industrial Research Institute for Automation and Measurements PIAP, 02-486 Warsaw, Poland; niuthon@gmail.com

**Keywords:** metallic glass, stress-impedance, GMI, Villari effect

## Abstract

The article presents the stress impedance investigation of CoFeNiMoBSi alloy in variation of the applied magnetic field. In order to carry out the study, a specialized stand was developed that allows for loading the sample with stresses and simultaneous action of the DC (direct current) magnetizing field. The tests were carried out for as-cast and Joule annealed samples. The significant influence of the magnetizing field acting on the sample on the stress-impedance results was demonstrated and the dependence of the maximum impedance change in the stress-impedance effect was determined, depending on the field acting. The obtained results are important due to the potential use of the stress-impedance effect for the construction of stress sensors.

## 1. Introduction

Since the discovery of the stress-impedance (SI) effect in amorphous alloys by Shen et al. [1], there has been ongoing research in both understanding and maximizing this effect from a material science point of view [2,3,4,5,6,7,8,9], and sensor development for practical engineering applications [10,11,12,13,14]. Soft magnetic amorphous materials, with positive and negative magnetostriction, exhibit a significant Villari effect, which alters the material’s magnetic permeability [15,16,17,18,19,20]. Due to the changes of magnetic permeability, the skin depth of the high-frequency current also changes, which leads to significant changes in the material’s impedance [21]. This effect constitutes the base of the SI phenomena.

The SI effect is, thus, a new addition to a broad series of magnetomechanical effects [22], some of which were first observed in the first half of the nineteen century, and are researched to this day due to high sensitivity of various experimental sensors, which can be obtained in newly developed materials [23,24,25,26,27,28,29,30,31,32]. The most known of the magnetomechanical effects are magnetostrictive and Villari effects [33], the latter leads to a change in magnetic permeability due to the mechanical stress, by inducing temporary magnetic anisotropy in stress direction [34].

The change of magnetic permeability is also significantly influenced by external magnetic fields, especially materials with very high magnetic permeability [35]. This, in turn, constitutes the base of giant magneto-impedance (GMI) effect [36,37,38]. While the GMI effect is heavily researched and there are many applications reported, it is detrimental for the SI effect, (potentially) significantly affecting the SI sensor output. However, most of the recent SI research is performed under ambient Earth field conditions, the value of which is rarely reported. It is worth noting that the SI effect, in turn, heavily modifies the GMI response and cannot be neglected [39], but it is much easier to take into account or even exploit in a sensor design stage [40].

Taking into account that both SI and GMI have (almost) the same underlying physical mechanism and the same influencing factors, but different expected measured variables, it is necessary to investigate the materials for SI applications with external magnetic field influence in mind. Ideally, one should find material with a strong magnetoelastic (Villari) effect, and a flat external magnetic field response, and, at the same time, maintains high magnetic permeability. These somewhat contradictory requirements can be potentially met in special class of GMI materials, having two peak characteristic with a wide plateau between GMI maxima due to perpendicular anisotropy [41]. Because it is a necessary (but not sufficient) condition, mainly due to nonlinearities of the Villari effect, the actual field dependence of SI characteristics should be experimentally investigated in search of most suitable material for SI sensors.

In the presented work, stress-impedance measurements in an amorphous ribbon under an external magnetic field is elucidated. A semi-automated measurement that is capable of precise measurements of SI characteristics, under the influence of an external DC magnetic field, is described. Effects of thermal treatment of the amorphous ribbon is given.

## 2. Materials and Methods

During the tests, ribbon samples were used. The investigated alloy Co_70_Fe_5_Ni_2_Mo_5_B_3_Si_15_ has a magnetostriction close to zero, and a high magnetic permeability of 100,000 [42]. The samples were prepared, with measurements of 60 mm long, 1 ± 0.1 mm wide, and 22 µm thick strips, by cutting from a spool of material. In order to precisely determine the stresses acting on the sample, the width was carefully measured using an optical micrometric microscope (PZO MWDC, Warsaw, Poland). The as-cast and Joule annealed samples were examined. The annealing was carried out with a current of 700 mA and 900 mA for a period of 1 h in a protective atmosphere of argon.

**Figure 1:**
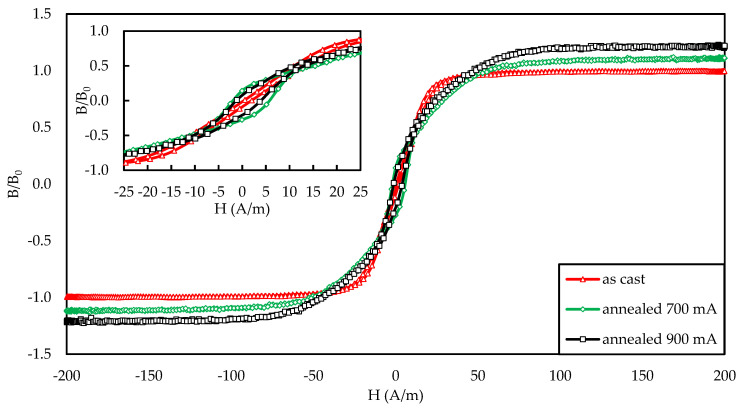
Hysteresis loop of as-cast and annealed samples used in the study.

Figure 1 presents the results of the magnetic property measurements of the research samples. The tests were performed using a hysteresis graph system (Blacktower Ferrograph, ESP, Warsaw, Poland) [43]. The B(H) induction plots were normalized to the B_0_ value of saturation of the as-cast sample.

**Figure 2:**
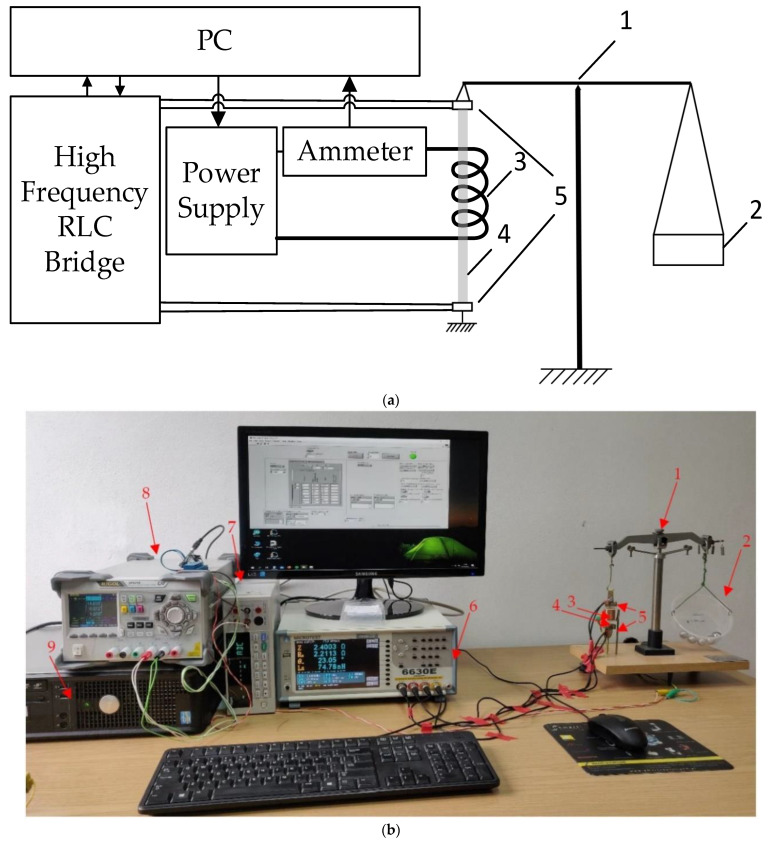
Developed test stand: (**a**) block diagram of the developed stand; (**b**) photo of the developed stand for testing stress-impedance in a function of the external magnetic field. The diagram and the photo contains: 1—balance scale, 2—applied mass, 3—magnetizing coil, 4—tested sample, 5—electrodes/measuring holders, 6—high-frequency RLC bridge, 7—ammeter, 8—laboratory power supply, 9—PC computer.

Figure 2a presents the block diagram of the SI test stand, and Figure 2b the photograph of the system. The impedance *Z* of the tested samples was measured using the four-wire method using a high-frequency RLC (Resistance, Inductance, Capacitance) bridge (Microtest 6630E, New Taipei City, Taiwan). The tests were carried out in the range of low and medium frequencies (<5 MHz) with a constant driving current *I_rms_* 10 mA.

The impedance change factor SI expresses the change in impedance with respect to minimum stresses *σ_min_*. It is calculated using the following Equation (1):

(1) $$\Delta Z/Z\left(\%\right)\;=\;100\;\cdot \;\frac{Z\left(\sigma \right)-Z\left(\sigma_{min}\right)}{Z\left(\sigma_{min}\right)}$$

The longitudinal tensile stresses, reaching up to about 200 MPa, were applied using a modified equal-arms laboratory scale. One of the sample holders was attached to the base, and the other was connected to the balance, so that the mass applied to the pan, attached to the other arm of the balance, applied tensile stress on a sample. The weight of the sample holder was compensated by putting special weights on the second arm of the balance, so that the initial external stresses in the sample were close to zero. The load was applied from the minimum value to the maximum with a constant step of 24.80 g. The value of the mass of a single weight was measured using an analytical balance (XA 82/220 3Y, Radwag, Radom, Poland). The gravity constant used for determining stresses g equal 9.81229 m·s^−2^ was taken from [44].

Stress-impedance measurements were made for a wide range of magnetizing fields acting on the sample. The coil provided a field in the range of ± 8000 A/m with a 10 A/m step. The coil was calibrated using a magnetometer (DSP 455, Lake Shore Cryotronics, Westerville, OH, USA) with calibrated hall probe (HSE, Lake Shore Cryotronics, Westerville, OH, USA), taking into account the Earth’s field. The value of the current flowing through the coil was set using a laboratory power supply (DP821A, Rigol Technologies Inc., Sha He Town, Beijing, China), and to precisely determine the field acting on the sample, the current was measured using an ammeter (TH1961, Changzhou Tonghui Electronic Co, Changzhou, China). A dedicated program for controlling the measurement system and collecting measurement data was developed by the authors using the National Instruments LabVIEW environment (v.17, National Instruments, Austin, TX, USA).

## 3. Results

**Figure 3:**
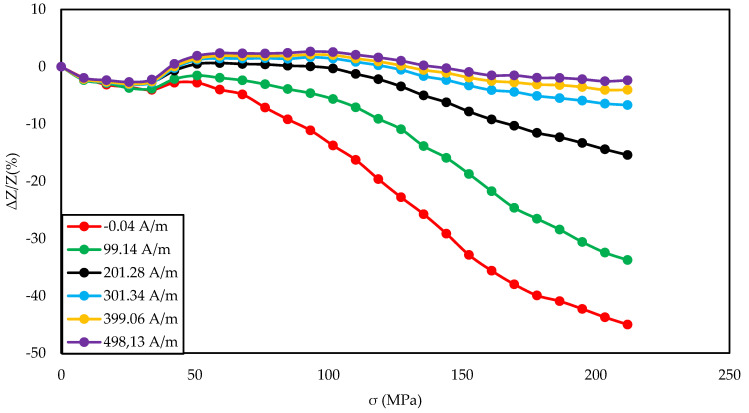
Dependence of impedance in a function of stresses for different magnetizing fields for the as-cast sample.

**Figure 4:**
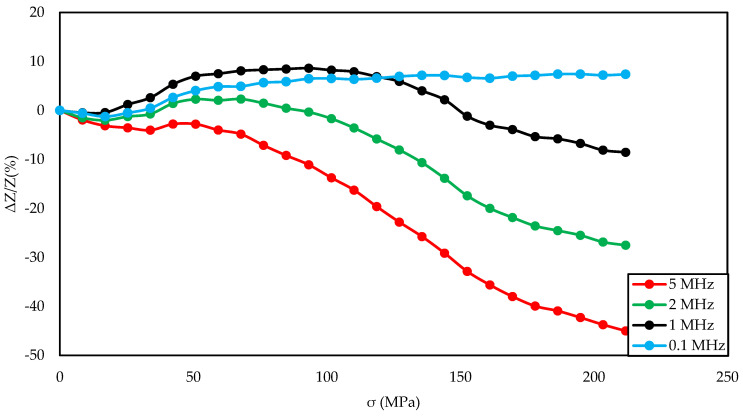
Dependence of impedance in a function of stresses for different driving frequencies for the as-cast sample and 0 A/m magnetizing field.

Figure 3, Figure 4, Figure 5, Figure 6, Figure 7, Figure 8, Figure 9, Figure 10 and Figure 11 present the results of the conducted experimental studies. For each of the prepared samples, graphs of longitudinal tensile stress dependence of the SI were prepared:

For range of magnetizing fields and the exciting frequency of 5 MHz (Figure 3, Figure 6, and Figure 9);

For set of frequencies and the magnetizing field for which the greatest impedance change was obtained (Figure 4, Figure 7, and Figure 10).

Additionally, graphs of the coefficient of the largest change in the impedance were prepared for a given magnetizing field according to the Equation (2):

(2) $$max\;\Delta Z/Z\;\left(\%\right)\;=\{\begin{array}{c} max\;\left(\Delta Z/Z\left(\sigma \right)\right),\;max\;\left(\Delta Z/Z\left(\sigma \right)\right)\geq \left|min\;\left(\Delta Z/Z\left(\sigma \right)\right)\right|\\ min\;\left(\Delta Z/Z\left(\sigma \right)\right),\;max\;\left(\Delta Z/Z\left(\sigma \right)\right)<\left|min\;\left(\Delta Z/Z\left(\sigma \right)\right)\right| \end{array}$$

**Figure 5:**
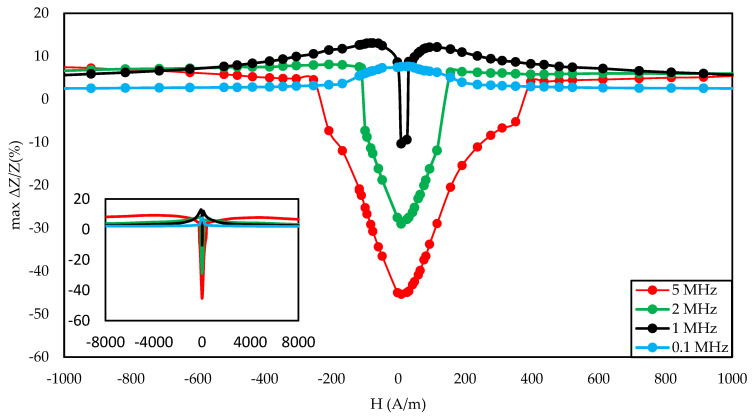
Dependence of max change of impedance ratio in a function of magnetizing field for different driving frequencies for the as-cast sample.

Figure 3, Figure 4 and Figure 5 show the SI for the as-cast ribbon sample. Figure 3 shows that, for stresses up to about 40 MPa, the influence of the magnetizing field was small. Then, for magnetizing fields below 300 A/m, the characteristic decreased, the smaller the value of the magnetizing field, the greater SI, reaching the maximum for the magnetizing field equal to 0 A/m. The highest SI value measured for this sample was −45.38%. Figure 4 shows that the frequency had an influence on the scale and the nature of impedance changed under stress. The greatest changes were recorded for the 5 MHz frequency, while the observed dependence decreased. With a decrease in frequency, a change in the characteristics was observed; for frequencies 2, 1, 0.1 MHz, an increase and stabilization of the characteristics can be distinguished, and the increase/stabilization was maintained in a wider range of stresses for lower frequencies. Max ΔZ/Z ratio (Figure 5) had a clear character of a single-peak curve, increasing the maximum value of the ratio with increasing frequency.

**Figure 6:**
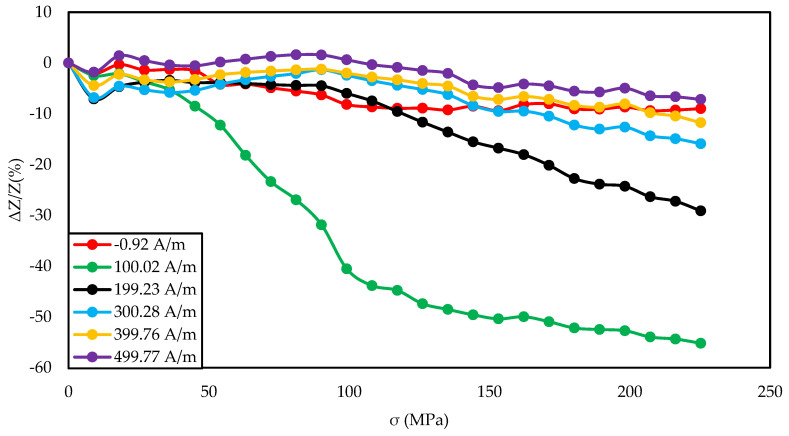
Dependence of impedance in a function of stresses for different magnetizing fields for the annealed by a 700 mA current sample.

**Figure 7:**
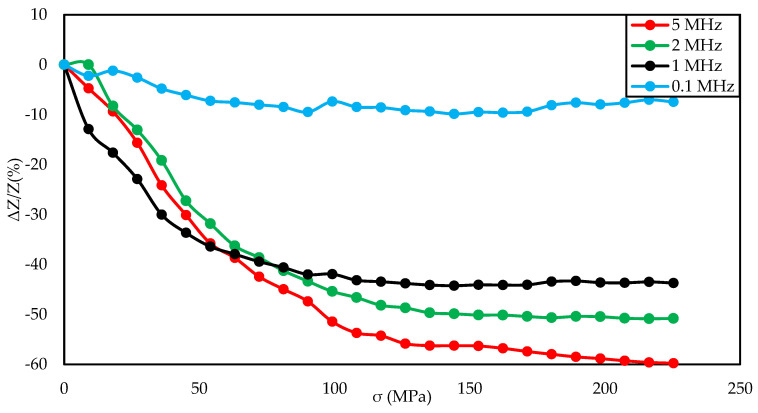
Dependence of impedance in a function of stresses for different driving frequencies for the annealed by a 700 mA current sample and −60 A/m magnetizing field.

**Figure 8:**
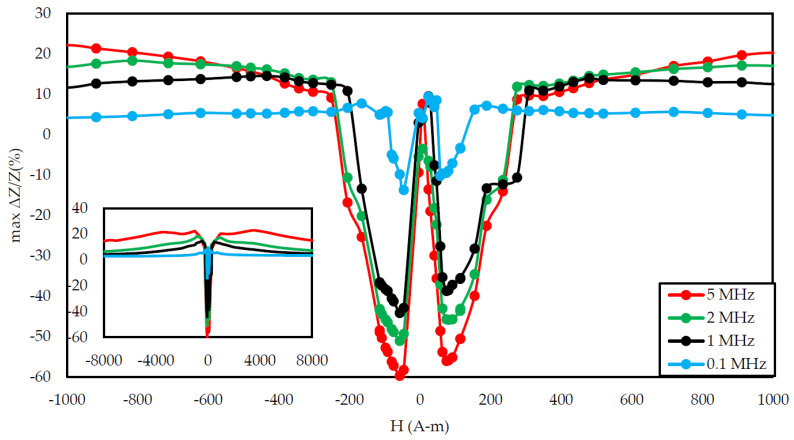
Dependence of max change of impedance ratio in a function of magnetizing field for different driving frequencies for the annealed by a 700 mA current sample.

In Figure 6, Figure 7 and Figure 8, the measurement results of a sample annealed with a current of 700 mA are presented. The diagram in Figure 6 shows that, for magnetizing fields close to 0 A/m and medium magnetizing fields (in the range of 300–500 A/m), the change in impedance under stress was small, below 10%. For the magnetizing field of 200 A/m, the characteristics were approximately constant for stresses up to 110 MPa, and then it decreased. In the case of the magnetizing field amounting to 100 A/m, the characteristic decreased sharply in the range from 20 to 120 MPa, while for higher stresses, the characteristics were: less sloping, slowly flattening. Figure 7 shows that the excitation frequency above 0.1 MHz does not affect the shape of the graph, but only the stress range in which the characteristic is decreasing and the maximum value of impedance change. For the frequency of 1,2,5 MHz, the plateau area was observed. The maximum SI value for this sample was −59.77%. The nature of the max ΔZ/Z ratio curve was clearly 2 peaks. The inset of Figure 8 presents a plot of the coefficient in the full tested magnetizing field, showing that the changes in the SI for 5 MHz occur in a very wide range of magnetizing fields.

**Figure 9:**
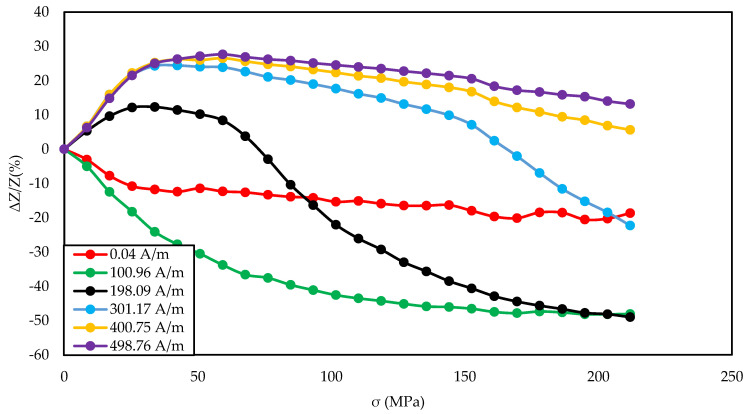
Dependence of impedance in a function of stresses for different magnetizing fields for the annealed by a 900 mA current sample.

**Figure 10:**
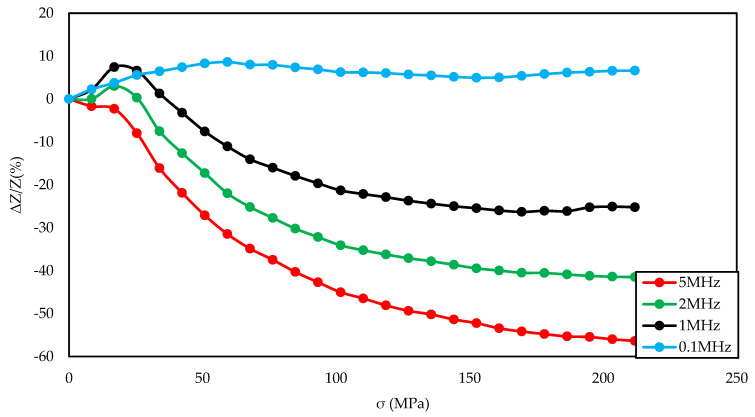
Dependence of impedance in a function of stresses for different driving frequencies for the annealed by a 900 mA current sample and −110 A/m magnetizing field.

**Figure 11:**
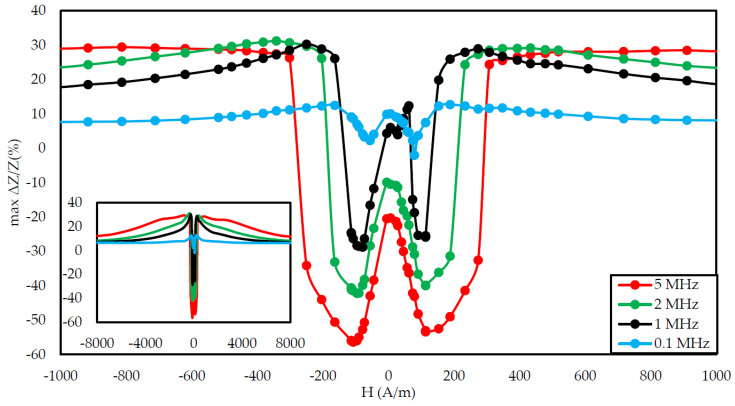
Dependence of max change of impedance ratio in a function of magnetizing field for different driving frequencies for the annealed by a 900 mA current sample.

Figure 9, Figure 10 and Figure 11 show the graphs for a sample annealed with 900 mA. For this sample, the nature of the impedance versus stress curve (Figure 9) depends much more strongly on the magnetizing field than for the as-cast sample (Figure 3) or the sample annealed with 700 mA current (Figure 6). For the near magnetizing field, there is a large slope of the characteristic in the range of 0–30 MPa, while in the later range, the changes are small. For a magnetizing field of approximately 100 A/m, the characteristic has a decreasing character in the entire range of operating stresses, and for larger fields, up to 30 MPa, the characteristic increases, followed by a slow and partly significant decrease. As the magnetizing field increases, the stress area for a small impedance drop increases. The highest SI value observed for this sample was −56.37%. In Figure 10, you can see that increasing the frequency causes greater changes in impedance. For the frequency of 1, 2, 5 MHz, a characteristic inflection point is visible at 20 MPa. It can be seen that the characteristic for the 1 MHz frequency is saturated, while for the remaining frequency values the saturation point was not reached. The graph in Figure 11 has a clear character of the 2-peak curve for all tested excitation frequencies.

## 4. Discussion

Numerous studies indicate a close dependence of the magnetic properties of amorphous co-based alloys on the stresses acting on the material [45,46]. However, a complete model of the dependence of magnetic permeability on mechanical stress has not been presented so far. The model that best describes this phenomenon was presented by Sablik et al. [47]. This solution uses the concept of the effective magnetic field *H_eff_*, acting on the sample, taking into account the variable and constant components of the acting magnetic field, mechanical stress, and others. According to the Jiles–Sablik model, *H_eff_* can be expressed as [16]:

(3) $$\vec{H_{eff}}\;=\;\vec{H}+\alpha M+\vec{H_{\alpha }}$$

where *H* is defined as the sum of the DC basing field and exciting AC (alternating current) magnetic field, α is dimensionless average filed associated with the interdomain coupling and *M* is the magnetization. The component of the effective field affecting the sample, related to mechanical stress-induced anisotropy field, is given by the relationship:

(4) $$\vec{H_{\alpha }}\;=\;\frac{3\lambda_{s}\sigma }{2\mu_{0}M_{s}}\frac{M}{M_{s}}$$

where *λ_s_* is the magnetostriction value in saturation, *µ_0_* is the magnetic permeability of the vacuum, and *M_s_* is the saturation magnetization. However, it is important that this model can only be used under small mechanical stress. In the range of high stresses that occurred in the test, the saturation magnetostriction *λ_s_* parameter is not constant. Moreover, it was also observed that for high values of mechanical stress, this parameter may change the sign [17].

Operation of stress on the sample changes the magnetic permeability of the sample [48]. This, in turn, changes the penetration depth *δ* of the exciting AC current in the stress-impedance effect according to the formula:

(5) $$\delta =\sqrt{\frac{\rho }{\pi \cdot f\cdot \mu_{0}\cdot \mu_{r}}}$$

where *ρ* is the resistivity of the material, *f* is the frequency of the AC current passed through the sample, *µ_0_* is the magnetic permeability of the sample, and *µ_r_* is the relative permeability. This formula is correct when using the SI (french. système international d’unités) system, which was used in this study. In the case of using the centimeter–gram–second (CGS) system, the relation proposed by Landau–Lifszyc [49] should be used.

The relationship between the ribbon impedance *Z* and the penetration depth *δ* is obtained by substituting the solution of Maxwell’s equations to the equation describing the impedance, using the surface impedance tensor (the transformation is fully described in [38,50]):

(6) $$Z=R_{DC}\cdot i\cdot \left(1+i\right)\cdot \delta \cdot a\cdot \coth(i\cdot \left(1+i\right)\cdot \delta \cdot a)$$

where *R_DC_* is the dc sample electrical resistance, *i* is an imaginary unit, and *2a* is the ribbon thickness.

Combining dependences 5 and 6, it can be seen that the change of the relative magnetic permeability of the material directly changes the impedance of the sample. Using the change in magnetic permeability resulting from the effective magnetic field *H_eff_* and the above dependence, it is possible to identify the influence of the acting magnetic field and stresses on the impedance of the tested sample. In a study, the change of the effective field *H_eff_* was influenced by the change of stresses (relationships 3 and 4), but also by the change of the field *H* acting on the sample during the stress-impedance test.

The graph in Figure 4 is clearly a single-peak curve, while those in Figure 8 and Figure 11 are a two-peak curve. These are the shapes characteristic of the study of the giant magnetoimpedance phenomenon [51], where the single-peak curve is obtained for samples with longitudinal anisotropy, and the two-peak curve for the transverse anisotropy (for the magnetizing field acting along the sample) [52]. The as-cast sample (Figure 3, Figure 4 and Figure 5) should have longitudinal anisotropy due to postproduction stress, and Joule annealed samples should have transverse anisotropy due to induced transverse field anisotropy (relative to the specimen). Therefore, for the as-cast sample, the SI changes will be the greatest for the 0 magnetizing field. Increasing the magnetizing field, acting on the sample, will block the motion of the domain walls and the rotation of the magnetization, which reduces the range of SI changes. For the sample annealed with the 700 mA current, the greatest changes are obtained for the magnetizing field in the range from 50 to 200 A/m, and for the 0 A/m field and above 200 A/m, the SI changes drop rapidly. This behavior of the sample is due to the fact that the greatest changes in SI are obtained for the situation in which the sample is affected by the field causing the sample’s magnetization along the axis of easy magnetization. Then the rotation of the magnetization vector and the movement of the domain walls is the most free; therefore, the SI changes are the greatest. We have a similar situation for a sample annealed with the area of 900 mA. However, for this sample, a larger area of maximum SI change was obtained.

The samples used in the study had nearly zero magnetostriction in the initial state [42]. Despite this, the study showed large stress-induced impedance changes. This is due to the significant change in saturation magnetostriction under stress. The performed Joule annealing process could also influence the value of saturation magnetostriction. The effect of stresses on the magnetostriction of amorphous alloys with a negligible magnetostriction saturation value has not yet been fully investigated. However, there are studies supporting this phenomenon [18].

## 5. Conclusions

The article presents previously unpublished research on the phenomenon of stress impedance in a wide range of magnetizing fields acting simultaneously on the sample. It has been shown that the change of the magnetizing field significantly changes the shape of the ΔZ/Z (σ) characteristic. This may, for example, lead to a change in the stress range for which the changes are significant and approximately linear, or the use of a field for which the changes are small (Figure 9). This opens up new possibilities in the construction of stress sensors based on the phenomenon of stress-impedance.

The conducted research shows that proper annealing significantly increases impedance changes in the stress impedance phenomenon. The performed annealing allowed to increase the SI coefficient by 32%. The largest SI changes, 59.77%, were achieved for the Joule annealed sample, with a current of 700 mA. For this sample, the greatest changes were in the range from 0 to 100 MPa, additionally, the characteristic was approximately linear.

## Data Availability

The data presented in this study are available on request from the corresponding author.
